# Assessing Locomotive Syndrome Through Instrumented Five-Time Sit-to-Stand Test and Machine Learning †

**DOI:** 10.3390/s24237727

**Published:** 2024-12-03

**Authors:** Iman Hosseini, Maryam Ghahramani

**Affiliations:** 1 School of Computing, Australian National University, Acton, ACT 2601, Australia; 2 Human-Centred Technology Research Centre, University of Canberra, Bruce, ACT 2617, Australia; maryam.ghahramani@canberra.edu.au

**Keywords:** inertial measurement unit, locomotive syndrome, machine learning, sit to stand

## Abstract

Locomotive syndrome (LS) refers to a condition where individuals face challenges in performing activities of daily living. Early detection of such deterioration is crucial to reduce the need for nursing care. The Geriatric Locomotive Function Scale (GLFS-25), a 25-question assessment, has been proposed for categorizing individuals into different stages of LS. However, its subjectivity has prompted interest in technology-based quantitative assessments. In this study, we utilized machine learning and an instrumented five-time sit-to-stand test (FTSTS) to assess LS stages. Younger and older participants were recruited, with older individuals classified into LS stages 0–2 based on their GLFS-25 scores. Equipped with a single inertial measurement unit at the pelvis level, participants performed the FTSTS. Using acceleration data, 144 features were extracted, and seven distinct machine learning models were developed using the features. Remarkably, the multilayer perceptron (MLP) model demonstrated superior performance. Following data augmentation and principal component analysis (PCA), the MLP+PCA model achieved an accuracy of 0.9, a precision of 0.92, a recall of 0.9, and an F1 score of 0.91. This underscores the efficacy of the approach for LS assessment. This study lays the foundation for the future development of a remote LS assessment system using commonplace devices like smartphones.

## 1. Introduction

The term “locomotive syndrome” (LS) was proposed by the Japanese Orthopaedic Association (JOA) in 2007 to define individuals who are prone to requiring nursing care services due to facing difficulties in performing activities of daily living (ADL) and hence are unable to live independently as the result of the physiological deterioration of locomotive organs [1]. The early diagnosis of any physical deterioration, specifically in such organs, is crucial to diminish the need for nursing care services [2]. The LS concept has thus attracted scientists’ and researchers’ attention around the world [3,4]. While LS was initially presumed to cause musculoskeletal disorder, several studies have also proved that certain psychological disorders result from LS [5,6].

The locomotive system is essential for mobility, and its health is particularly critical for older people. Over the past 40 years, there has been a notable increase in chronic diseases affecting the locomotive organs among middle-aged and older individuals, leading to a higher demand for surgical interventions [7,8]. Key issues include acute pain exacerbating mobility disturbances, complications from surgeries in patients with severe osteoporosis [9,10], and the significant influence of preoperative mobility on postoperative outcomes [11]. Moreover, delays in hospital discharge are increasingly common due to the longer rehabilitation needs of older patients. Locomotive syndrome refers to the decline in mobility functions, significantly affecting older adults’ independence and quality of life [12]. It is crucial to understand and address this syndrome to enhance the wellbeing of the aging population and ensure they maintain their mobility and independence.

The early diagnosis of LS followed by physical exercise training has been promoted by the JOA in order to improve QOL. The 25-question Geriatric Locomotive Function Scale (GLFS-25) was proposed in 2012 to assess LS and screen for declines in motor performance [13]. The GLFS-25 questionnaire is a self-administered questionnaire, which consists of 25 questions in six latent factors, i.e., body pain, movement-related difficulty, usual care, social activities, cognitive status, and daily activities [14]. Individuals score their performance qualitatively on the five-point scale. In this case, each question is graded from 0 (i.e., no impairment) to 4 (i.e., severe impairment). The assessed grades of all the questions are added together to indicate the severity of the locomotion. That is, a higher total score corresponds to worse mobility function. According to their overall score, participants are classified into three groups, namely Non-LS (score <7), LS-Stage 1 (7≤ score <16), and LS-Stage 2 (score ≥16) [15]. LS-Stage 1 indicates a deterioration in locomotive organs, whereas participants classified as LS-Stage 2 are at high risk of not living independently.

Several physical performance tests have been developed in which participants take part in a series of activities to assess their motor functions. The two-step test, stand-up test, single-leg stand, hand-grip test, and timed-up-and-go (TUG) test have been proposed and analyzed [16,17,18]. However, the proposed methods are qualitative, subjective, resource-intensive and require a long time to complete, and the response rate is reportedly low in older people [19]. Research shows clear connections between frailty, risk of falling, and locomotive syndrome (LS). Older adults with higher fall risk scores tend to have higher GLFS-25 scores, and those with LS are more likely to fall [20,21]. Despite their overlaps, frailty, risk of falling, and LS are distinct. Frailty indicates a high risk of severe outcomes like death and disability, while LS, which affects those with musculoskeletal issues, predicts future mobility problems but not necessarily falls [22].

Sit-to-stand (STS) tests are effective for assessing these conditions. The STS movement is one the most common and physically demanding daily activities, requiring coordination and balance [23,24]. Kataoka et al. [25] assessed the lower limb kinematics of subjects with LS using IMUs. It was seen that participants with Stage 1 and Stage 2 LS had significantly smaller hip angular velocity and knee extension during their sit-to-stand (STS) compared to the control group. Their results suggest the need for STS movement training to prevent LS-Stage 1 in the elderly. The five-time sit-to-stand (FTSTS) test, often used in clinical settings, measures lower limb strength and fall risk [26,27]. Studies have shown that instrumented FTSTS tests with wearable sensors provide valuable insights into the biomechanics of this movement and are good indicators of risk of falling [28,29,30].

Many studies have focused on the integration of machine learning and instrumented FTSTS for the prediction of risk of falls or monitoring the progression of various disorders. Park et al. [31] conducted a study utilizing the instrumented FTSTS in combination with machine learning techniques for frailty assessment in older adults. Yang et al. [32] explored the viability of using machine learning and instrumented FTSTS to assess the impact of short-term rehabilitation in poststroke patients. In another study, FTSTS and machine learning were used for knee osteoarthritis detection [33]. Due to its capability to measure lower body strength, balance, and mobility, when instrumented and coupled with machine learning, the FTSTS becomes a powerful tool for health assessment and personalized interventions.

Machine learning and deep learning have emerged as transformative tools for analyzing complex datasets, particularly in healthcare and mobility assessment domains due to their capacity to process large volumes of data and extract meaningful patterns. These methods enable the extraction of non-linear patterns and relationships from large-scale data, which are often challenging to identify through traditional statistical approaches. By leveraging these computational techniques, researchers have advanced the understanding and prediction of various health conditions. In this case, machine learning models have been employed to monitor and predict health outcomes in diverse applications such as chronic diseases and conditions associated with aging. For instance, both traditional machine learning and deep learning models, in combination with data recorded from IMUs, have been widely used for frailty analysis [34] and fall risk assessment [35], which highlights their abilities to extract meaningful insights from sensor-based datasets. Similarly, wearable devices equipped with heart rate and blood pressure sensors have used random forest and gradient boosting machine to predict cardiovascular events [36,37]. Moreover, deep learning models, such as recurrent neural networks and long short-term memory networks, have been extensively used to model sequential patterns in glucose levels for patients with diabetes, enabling the prediction of hypo- or hyperglycemic events [38,39]. Ensemble neural networks have also been used to predict treadmill walking energy expenditure in a contactless system, which plays an important role in health evaluations, particularly in older adults [40]. These applications highlight the ability of machine learning and deep learning models to analyze time-series data in real time, providing actionable insights into various healthcare domains.

This study for the first time assessed the feasibility of using the instrumented FTSTS test and machine learning to evaluate LS. In our previous study, we explored the feasibility of identifying older individuals with LS through the use of an instrumented FTSTS test [41]. Various features from the FTSTS data were extracted and significant differences in a set of these features between non-LS and LS older individuals were observed. Building upon this foundational research, our current study represents a continuation of our efforts. To the best of our knowledge, this study is the first to use an instrumented functional test and machine learning for the classification of participants in different stages of LS. Through this innovative approach, this study offers the possibility of the early quantitative assessment and remote evaluation of LS, ultimately leading to more timely and effective interventions. While frailty and fall risk have been studied, our unique emphasis on LS highlights its distinct and potentially more alarming implications, making a significant contribution to the field.

**Figure 1:**
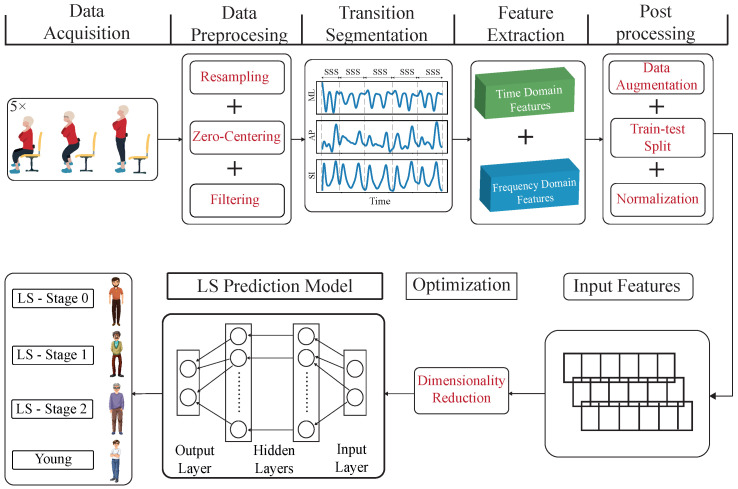
Overview of the study stages. This figure illustrates the comprehensive workflow for assessing locomotive syndrome (LS) using an instrumented five-time sit-to-stand (FTSTS) test and machine learning techniques in order to predict and identify the different stages of LS. The process begins with data acquisition, followed by data preprocessing steps, including zero-centering, filtering, and data augmentation. Sit–stand–sit (SSS) transition segmentation and postprocessing were then applied to the data. Feature extraction involved generating both time-domain and frequency-domain features, which were subsequently optimized and underwent dimensionality reduction. The data were then split into training and testing sets, then normalized before being input into the machine learning model. The model consists of an input layer, multiple hidden layers, and an output layer, which classifies the data into different stages of locomotive syndrome (LS-Stage 0, LS-Stage 1, LS-Stage 2).

In this study, a group of healthy younger participants and a group of older participants were recruited, with the latter categorized into LS stages 0–2 based on their GLFS-25 questionnaire. All participants were fitted with a single IMU at the pelvis level to record accelerometer and angular velocity data during the test. Various time and frequency domain features were extracted from the data, which were then utilized to train multiple machine learning models. The analysis and performance of these models were compared, revealing that multilayer perceptron exhibited the highest accuracy in classifying LS stages. Ultimately, this research project aimed at setting the basos for a larger study that could help with easy and remote LS assessment in older people. Figure 1 illustrates the comprehensive workflow employed for assessing locomotive syndrome through an instrumented FTSTS test coupled with machine learning techniques. We started by collecting data using a single IMU while participants performed the FTSTS test. The data were then preprocessed through resampling, zero-centering, and filtering to reduce noise and ensure consistency. After preprocessing, we segmented the data into three key transitions: sit-to-stand, stand-to-sit, and sit-to-stand-to-sit. This segmentation was essential as each phase represents distinct biomechanical characteristics and movement dynamics crucial for detecting subtle changes related to LS. From each segment, we extracted a variety of features in the time and frequency domains across three dimensions. These features allowed us to capture different aspects of movement and postural control. We addressed class imbalance through postprocessing techniques like data augmentation to improve model robustness. Following this, we performed a train–test split, applied normalization, and optimized the feature set with dimensionality reduction. This feature-based approach was chosen due to our limited sample size, which made deep learning less feasible at this stage. Extracting features ensured reliability and interpretability, avoiding overfitting and leveraging domain-specific insights. As we expand our dataset, we plan to incorporate deep learning for more comprehensive raw data analysis in the next phase. Each stage of the study is discussed in detail in the Methodology Section.

## 2. Methodology

### 2.1. Participants

For this study, a total of 154 older participants, aged 60 and above, and 52 younger participants, aged between 18 and 45 years, were recruited to participate in the experimental tests. This study was approved (project number: 9208) by the University of Canberra Human Research Ethics Committee. Participants with severe comorbidities that could significantly impair motor function, such as Parkinson’s disease, dementia, or other major neurological disorders, were excluded to prevent confounding factors that could affect the study outcomes. Moreover, participants with severe musculoskeletal issues affecting their sit-to-stand postural transition were also excluded. This included individuals with significant arthritis, joint deformities, or other conditions that could hinder their ability to perform the movement effectively. Additionally, individuals who were unable to perform the FTSTS test independently were also excluded. This criterion was important to ensure that all participants could reliably complete the test without assistance, enhancing the integrity of our data collection and allowing for a more accurate assessment. All participants read and signed an informed consent statement. Additionally, the older participants were requested to complete the GLFS-25 questionnaire. Data from 28 older participants and 2 younger participants were excluded from the analysis. Out of the total 30 excluded participants, 6 were due to data transmission failure, and 24 were unable to perform all transitions in the FTSTS. The final analysis included participants who met the following recruitment criteria: (a) independent ability to perform the FTSTS test, (b) comprehension of instructions, and (c) completion of the questionnaire and consent form. Based on their total score on the questionnaire, older participants were then categorized into three groups: Non-LS (with a score less than 7), LS-Stage 1 (with a score between 7 and 15), and LS-Stage 2 (with a score of 16 or higher).

In this study, both younger and older participants were included deliberately. This decision was driven by the intention to distinguish between younger individuals and those classified as Stage 0 in the older age groups. The initial staging was based on the GLFS-25 questionnaire, and it is crucial to acknowledge the potential ceiling effect associated with questionnaire-based methods. This choice was made to specifically capture and differentiate the characteristics of younger individuals and those in the initial stage, providing a more nuanced understanding of LS across different age groups. Table 1 shows a summary of the participants’ demographic information including the number of participants, sex, and mean age in different classes.

### 2.2. Experimental Test and Protocol

Participants were asked to fully stand up arm-folded from a chair with a standard height of 40 centimeters five times consecutively at their own convenient pace. During the tests, participants were asked to try to maintain their balance without any extra movements, look straight ahead, stand up completely (i.e., full extension) and sit down (i.e., touch the chair) between each repetition. For the older participants in case of failure, a second trial was performed with a 10 min rest between the trials.

### 2.3. Data Collection and Preprocessing

An IMU (MTw from Xsens Technology, Enschede, Netherlands), equipped with a 3-axis gyroscope and a 3-axis accelerometer, was attached to the trunk at pelvis level in the lumbar region of the back (between L2 and L3 vertebrae) of the participants. Some researchers, such as Najafi et al. [42], have used a chest-level placement of the IMU, while others have opted for placement on the lower back [43,44]. Lower-back placement has proven to be the most practical and effective. It minimizes participant discomfort and reduces hardware complexity, making it the preferred choice for FTSTS analysis [45,46]. The MTw system features advanced built-in calibration and validation through its Strap Down Integration algorithm, Awinda protocol for real-time packet loss handling, and the Xsens Kalman Filter for accurate orientation estimates [47]. The angular rotation of the pelvis in the sagittal plane between the horizontal axis and the vertical axis (parallel to the subject’s spine) was recorded. The sampling rate used was 100 Hz, and the data were transmitted to a computer for analysis using Python. A preprocessing step known as zero-centering, which involves subtracting the mean value of each signal from the recorded data, was utilized. This method effectively eliminates any constant offsets that may distort the analysis. This helped us focus on the dynamic changes in acceleration rather than being influenced by any offset. Following zero-centering, we analyzed the variations in acceleration on the three axes. This allowed us to capture the subjects’ movement dynamics, even if they leaned or shifted during the tests.

The recorded data from the IMU underwent resampling to a frequency of 50 Hz. Subsequently, a 3rd-order Butterworth low-pass filter was applied to the data using a cut-off frequency of 5 Hz. This filtering process helped to reduce high-frequency noise and disturbances, resulting in a smoother and more reliable dataset for further analysis and interpretation.

### 2.4. Transition Segmentation

**Figure 2:**
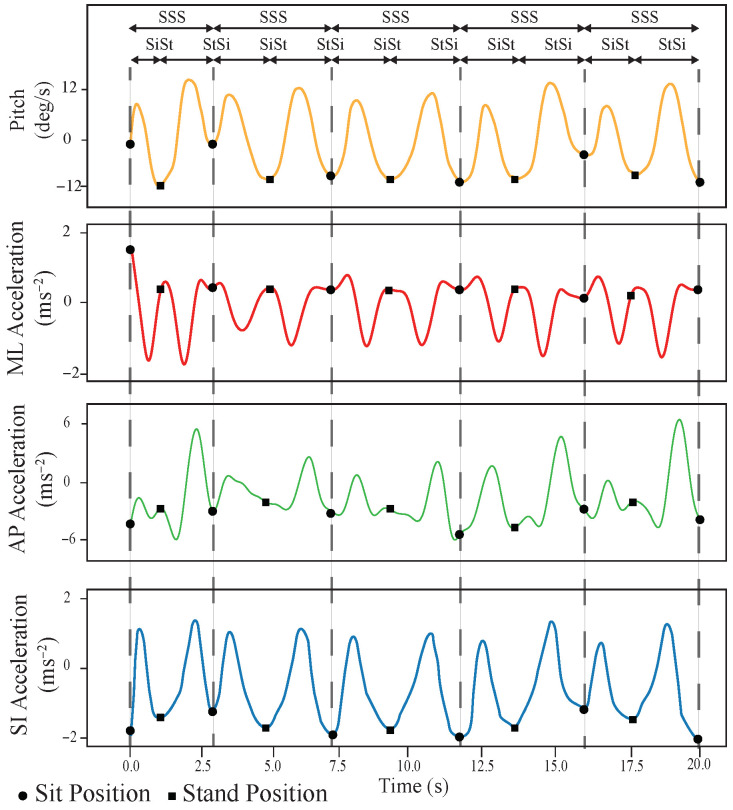
The acceleration of the pelvis in the ML, AP, and SI directions for a younger participant. The SiSt, StSi, and SSS transitions during the FTSTS test are shown.

In the analysis of the FTSTS experiment, the segmentation into distinct phases such as sit–stand (SiSt), stand–sit (StSi), and sit–stand–sit (SSS) transitions is crucial for understanding the dynamics of the activity. This transition segmentation was conducted to capture the specific biomechanical and movement dynamics associated with each transition phase of the FTSTS test. Each mode represents a unique aspect of postural control, lower limb strength, and coordination, which are critical for assessing the locomotion of participants. For instance, the SiSt postural transition is widely used to evaluate lower limb function, strength, and balance in older adults [48]. The performance on Sthe iSt transition has been linked to leg extensor strength, vestibular impairments [49], and alterations in movement strategies [50]. As a result, SiSt and StSi transitions have been applied in various contexts, including the evaluation of postural control [51], fall risk [42], poststroke impairments [52], and overall disability [53]. The SSS transition combines the elements of both phases and represents the ability to perform repetitive transitions efficiently. By segmenting the data, we ensured that the nuanced characteristics of each transition were preserved, enabling a more detailed analysis of how these phases relate to different LS stages. This segmentation also facilitated the extraction of phase-specific features, which are crucial for developing accurate predictive models. This segmentation ws accomplished using change in pitch data recorded on the gyroscope of the IMU, which represents the forward and backward tilt of the IMU. To identify the transitions, we focused on detecting the local minima in the change in the pitch data, as these points correspond to critical phases in the movement [54]. By leveraging the indices associated with these local minima, we could precisely pinpoint the moments when a participant transitioned between sitting and standing, standing and sitting. The recorded trunk accelerations of a young participant along the mediolateral (ML), anterior–posterior (AP), and superior–inferior (SI) directions are presented in Figure 2. Additionally, it highlights the corresponding transitions between the SiSt, StSi, and SSS segments during the FTSTS test.

### 2.5. Feature Extraction

In a study conducted by Doheny et al. [55], time- and frequency-domain features were extracted from SiSt, StSi, and SSS; total FTSTS transitions were extracted to predict fall risk. Consistent with their approach, we extracted the following quantitative metrics from the accelerometer data in both the time and frequency domains for each participant during the FTSTS, SiSt, StSi, and SSS transitions across the ML, AP, and SI planes, as well as the resultant acceleration defined in Equation (1).

(1) $$Resultant=\sqrt{Acc_{ML}^{2}+Acc_{AP}^{2}+Acc_{SI}^{2}}$$

The resultant acceleration is a composite measure derived from the accelerations in the ML, AP, and SI planes. This will provide us with a holistic view of the body’s movement during transitions in order to capture the combined effect of these three directional accelerations.

The following measures were calculated with respected to the acceleration. In this case, the variable *x* works as a place holder to represent the acceleration in the ML, AP, and SI directions, as well as the resultant acceleration across these planes. Specifically, $Acc_{x}$ refers to acceleration in one of these individual directions at a time.

**Time-Domain Measures**:

1. Transition duration (*s*): The transition duration represents the time taken to complete each transition, which is crucial for assessing the fluidity and efficiency of the movement, and is calculated by dividing the length of the acceleration data ($Acc_{x}$) by the corresponding sampling frequency (50 Hz after preprocessing):
  (2) $$T=\frac{Length(Acc_{x})}{Frequency}$$
2. Maximum acceleration ($\frac{m}{s^{2}}$): This metric captures the highest magnitude of acceleration achieved during each transition, which shows the intensity of the movement.
3. Range of acceleration ($\frac{m}{s^{2}}$): The range of acceleration on the *x* axis reflects the variability in movement and is determined by calculating the difference between the maximum and minimum values of $Acc_{x}$:
  (3) $$Range_{x}=max\left(Acc_{x}\right)-min\left(Acc_{x}\right)$$
4. Root mean squared (RMS) of the acceleration ($\frac{m}{s^{2}}$): The RMS value provides a measure of the overall magnitude of the acceleration signal, which is useful for assessing the average intensity of the movements. It represents the square root of the average of the squared accelerations on the *x* axis. The RMS equation used here was adapted from [56] and calculated as
  (4) $$RMS_{x}=\sqrt{\frac{1}{length(Acc_{x})}\sum_{i=1}^{3}Acc_{x_{i}}^{2}}$$
5. The maximum value of the resultant acceleration magnitude ($A_{Max}$): The maximum resultant acceleration highlights the peak combined acceleration across all planes, which can be critical for understanding the most intense phase of the transition and is calculated as
  (5) $$A_{Max}=\max\left(\frac{\sqrt{\sum_{i=1}^{3}{\left(Acc_{x_{i}}\right)}^{2}}}{length(Acc_{x})}\right)$$
6. Jerk ($\frac{m^{2}}{s^{5}}$): Jerk is a measure of the rate of change in acceleration and quantifies the smoothness of the transition by measuring the rate of change of acceleration, providing insights into the control and stability of movements. Specifically, the sway jerk of the acceleration on the *x* axis is computed as the average squared difference between consecutive $Acc_{x}$ values, divided by the transition duration (*T*):
  (6) $$Jerk_{x}=\frac{1}{T}\sum_{i=1}^{N-1}{\left|Acc_{x_{i+1}}-Acc_{x_{i}}\right|}^{2}$$
  *N* and *T* represent the number of samples used for each calculation and test duration, respectively.

**Frequency-Domain Measures**:

1. Total power: Total power is a measure of the overall energy contained in the acceleration signal on the *x* axis and represents the cumulative energy of the acceleration signal, which is essential for understanding the overall dynamic activity during transitions. It is calculated by integrating the power spectrum obtained through the Fourier transform (F):
  (7) $$TotalPower_{x}=\sum_{i=1}^{N}\left|F{\left(x_{i}\right)}^{2}\right|$$
  where F represents the Fourier transform.
2. Spectral edge frequency 50 (SEF50): This metric denotes the frequency at which half of the signal’s power is concentrated, which defines the dominant frequency components of the movement.
  (8) $$SEF50_{x}=\mathrm{Median}\{f:\sum_{i=1}^{N}|F\left(x_{i}\right){|}^{2}\leq 0.5\sum_{i=1}^{N}|F\left(x_{i}\right){|}^{2}\}$$
3. Spectral edge frequency 95 (SEF95): SEF95 is used to determine the frequency at which the majority of the signal’s power is concentrated, helping to identify the primary movement frequencies.
  (9) $$SEF95_{x}=\mathrm{Median}\{f:\sum_{i=1}^{N}|F\left(x_{i}\right){|}^{2}\leq 0.95\sum_{i=1}^{N}|F\left(x_{i}\right){|}^{2}\}$$
  In both Equations (8) and (9), $F(x_{i})$ represents the Fourier transform of the acceleration signal at frequency $x_{i}$, and *N* represents the total number of frequency components in the spectrum.

For each particular test, the mean and coefficient of variance (CV) of each of SiSt, StSi, and SSS were also explored.

The combination of these features in both the time and frequency domains generated a total of 144 distinct metrics. These features encompassed a comprehensive representation of the analyzed data, providing a rich set of information for further analysis and modeling.

### 2.6. Feature Optimization

Feature optimization is pivotal in model analysis, significantly impacting overall performance. The inclusion of irrelevant features within the dataset can impact a model’s efficiency. Therefore, proficient feature selection techniques are essential to maximize the model’s potential. In this case, we incorporated principal component analysis (PCA) into the whole dataset before splitting the dataset into train and test sets. Through PCA, the original feature matrix was transformed into a lower-dimensional representation while preserving the most relevant information. By projecting the data onto a new set of orthogonal axes, PCA helps with reducing the dimensionality of the feature set while improving the model’s performance. This leads to more efficient training processes, with a reduced set of features in use.

Furthermore, the feature selection process was augmented by employing the SelectKBest method [57] with mutual information, as provided by the scikit-learn library. This method assigns individual scores to all features within the feature set, offering valuable insights into their importance in predicting the target outcomes. Features with higher scores are considered more influential, further contributing to the overall optimization of the model’s predictive capabilities. Together, these strategies ensure the model is equipped with the most pertinent features, maximizing accuracy and efficiency in analyzing a large set of extracted features. In this case, mutual information method [57] was used to score features based on their predictive relevance. By focusing on the most significant features, we were able to enhance our model’s ability to generalize while ensuring efficient and accurate analysis with a reduced set of highly relevant features.

### 2.7. Train, Test, and Data Preprocessing

The data were split into training and test sets with a 20% test size used for the split after data preparation. The random state was set to 42 throughout the analysis to achieve a consistent split when comparing results. The training set was expressly reserved for training the model, enabling the learning algorithm to gain insights from the available data. On the other hand, the test set played a crucial role in evaluating the trained model’s performance on unseen data.

The z-score normalization from the scikit-learn library was used to prevent potential information leakage from the test set into the training set as well as to more accurately simulate a real-world scenario, where the model encounters unseen data during testing. By assessing how well the model generalized to new instances, its effectiveness in potential real-world situations was gauged.

In cases like ours, where there is an under-representation of the minority class (LS-Stage 1), data augmentation becomes crucial for handling imbalanced datasets. To address this issue, the synthetic minority oversampling technique (SMOTE) was employed [58]. This technique generates synthetic samples strategically placed between the existing samples of the minority class (LS-Stage 1), effectively creating a more balanced training dataset. This technique generates synthetic samples by interpolating between existing samples of the minority class, addressing class imbalance by expanding the representation of this class without duplicating exact samples. This interpolation is performed along the line segment connecting a sample to one of its nearest neighbors within the minority class, as noted in [59]. This approach preserves the statistical properties of the minority class, aiming to reduce potential bias and enhance the model’s ability to generalize across classes. However, as Elreedy and Atiya discussed in [60], SMOTE-generated samples may sometimes create clusters of minority samples along the boundary between classes, which can slightly shift the decision boundary in favor of the minority class, which can potentially impact the classification boundary. This trade-off is particularly useful for classifiers that are prone to majority class bias, which can ultimately improve the performance on imbalanced datasets.

The labels associated with the dataset are transformed into a suitable representation for multiclass classification tasks. Specifically, the labels are converted into a one-hot encoding format. One-hot encoding translates the categorical labels into binary vectors, where each class corresponds to a binary column. This representation facilitates the model’s ability to classify instances into their respective classes accurately and efficiently. See Algorithm 1.
**Algorithm 1** Feature selection and data preprocessing.**Require:** Raw data **Ensure:** Preprocessed data with selected features
  1:Load raw data  2:Apply SMOTE to balance the dataset  3:Split data into training and testing sets (80/20 split)  4:Normalize data using z-score normalization  5:Apply principal component analysis (PCA)  6:   Convert the labels to one-hot encoding  7:   Compute covariance matrix  8:   Compute eigenvalues and eigenvectors  9:   Select top *k* eigenvectors10:   Transform data to new feature space11:(Alternatively) Apply SelectKBest with mutual information12:   Compute mutual information for each feature13:   Rank features based on mutual information14:   Select top *k* features15:**return** Preprocessed data

### 2.8. Classification Experiments

#### 2.8.1. Deep Learning Approaches

We used a multilayer perceptron (MLP) deep learning model using the sequential API of the Keras library in Python. The MLP model was selected over others due to its superior performance in applications of human activity recognition, especially in the assessment of risk of falls in older people [61,62,63,64,65]. The MLP’s ability to capture complex nonlinear relationships in the data recorded from accelerometer and gyroscope [66,67], coupled with its flexibility in tuning various hyperparameters, made it the most effective model for our study. Our MLP model comprises five hidden layers, each applying the rectified linear unit (ReLU) activation function, and a final output layer utilizing a SoftMax activation function for multiclass classification. The input dimensionality, which is equivalent to the number of features, enters the first dense layer with 512 neurons. Each subsequent hidden layer halves its number of neurons, from 256 in the second layer to 128, 64, and finally 32 neurons in the fifth layer. This design strategy is commonly employed to gradually condense the information from the input space to the output space, mimicking an information funnel. To prevent overfitting in our ML model, we implemented L2 regularization and incorporated early stopping measures. To identify the optimal combination of hyperparameters, an extensive grid search was conducted using the Keras Tuner library, systematically exploring a range of values for the parameters as follows:

- Learning rate: [0.1,0.01,0.001,0.0001];
- Number of epochs: [100,200,300,…,1000];
- Batch size: [16,32,64,128]
- L2 regularization: [0.01,0.001,0.0001];
- Early stopping: [True, False];
- Optimizer: [‘sgd’, ‘adam’].

#### 2.8.2. Traditional Machine Learning Approaches

Alongside the deep learning approach, several traditional machine learning models were employed. All the extracted features were utilized as inputs for these models. In order to optimize the performance of the different classification methods, grid search cross-validation (GridSearchCV) was employed for hyperparameter tuning [68]. GridSearchCV systematically navigates hyperparameter tuning by establishing a grid of potential values. This approach exhaustively explores the parameter possibilities within the grid, aiding in pinpointing the most suitable hyperparameters for each machine learning method. The machine learning methods compared in this section include K-nearest neighbors (KNN), support vector machine with linear and radial basis function kernels (SVM-lin, SVM-rbf), decision trees (DTs), random forest (RF), gradient boosting classification (GB), and logistic regression (LR). The choice of these traditional machine learning models was based on their proven effectiveness in classification tasks, particularly in the context of biomechanical data analysis and their varying mechanisms for learning from data. A summary of the grid search can be found in Table 2. This table outlines the parameter ranges explored during the grid search for each machine learning model.

The choice of these parameters was guided by their known impact on model performance and their ability to capture the variability in the data effectively. For example, the regularization parameter (‘C’) in SVM helps to avoid overfitting by balancing the trade-off between achieving a low training error and minimizing the complexity of the model. The gamma parameter in SVM with RBF kernel controls the influence of individual training examples, with low values meaning ‘far’ and high values meaning ‘close’. In the KNN model, the number of neighbors (n_neighbors) directly impacts the decision boundary and the smoothness of the model. The weight parameter in KNN can be set to ‘uniform’, where all points in each neighborhood are weighted equally, or ‘distance’, where closer neighbors of a query point have a greater influence than neighbors that are further away. The max_depth parameter in DT limits the depth of the tree to prevent overfitting, while the criterion parameter determines the function to measure the quality of a split (e.g., ‘gini’ for the Gini impurity and ‘entropy’ for the information gain). For the RF model, n_estimators refers to the number of trees in the forest, max_depth sets the maximum depth of the tree, min_samples_split is the minimum number of samples required to split an internal node, and min_samples_leaf is the minimum number of samples required to be at a leaf node. The bootstrap parameter indicates whether bootstrap samples are used when building trees. In GB model, learning_rate shrinks the contribution of each tree by learning_rate, max_depth limits the depth of the trees, min_samples_split and min_samples_leaf are the same as in random forest, and n_estimators is the number of boosting stages to be run. For LR model, the regularization parameter (C) controls the strength of the regularization, with smaller values specifying stronger regularization. The penalty parameter specifies the norm used in the penalization (e.g., ‘L1’ for Lasso regression and ‘L2’ for Ridge regression). See Algorithm 2.
**Algorithm 2** Training of machine learning models.**Require:** Preprocessed training data **Ensure:** Trained models
  1:Define parameter grid for each model  2:**for** each model in {SVM, KNN, DT, RF, GB, LR, MLP} **do**  3:   Initialize model with default parameters  4:   Perform GridSearchCV to find optimal hyperparameters  5:   **for** each combination of parameters in the grid **do**  6:     Train model using cross-validation  7:     Evaluate model performance  8:     Select combination with best performance  9:   **end for**10:   Train final model using optimal hyperparameters on full training data11:**end for**12:Save trained models

### 2.9. Performance Evaluation

Various evaluation metrics were utilized to comprehensively assess the machine learning models’ performance in LS classification on both the training and testing datasets. To thoroughly explore the impact of different factors and the contribution of various feature sets, multiple models were trained using diverse combinations of features and hyperparameters.

To attain a nuanced comprehension of model effectiveness, some evaluation metrics such as accuracy, precision, recall, and the F1 score were measured. Precision quantifies the proportion of true positive predictions among all positive predictions, while recall measures the ability of the model to identify all positive instances.

By considering these evaluation metrics on both the training and testing datasets, we assessed the model’s performance in different scenarios and gauged its ability to generalize to unseen data. This comprehensive evaluation enabled us to make informed decisions about the model’s effectiveness and suitability for the classification of locomotive syndrome. See Algorithm 3.
**Algorithm 3** Evaluation of models.**Require:** Trained models, testing data **Ensure:** Evaluation metrics (accuracy, precision, recall, F1 score)
  1:Load testing data  2:**for** each model in {SVM, KNN, DT, RF, GB, LR, MLP} **do**  3:   Make predictions on testing data  4:   Compute evaluation metrics:  5:      Accuracy  6:      Precision  7:      Recall  8:      F1 score  9:**end for**10:Compare performance of models11:**return** Evaluation metrics

## 3. Results and Discussion

In this study, we used machine learning with an instrumented FTSTS test to assess LS stages, aiming for a more accessible and dynamic evaluation using a single, widely available IMU. This approach provides a simpler, more inclusive alternative to previous machine learning methods for LS detection, which often depend on complex or specialized equipment. For example, Takahashi et al. [69] developed a convolutional neural network model to estimate the probability of LS based on foot pressure images; however, this method requires specialized gait pressure equipment. Similarly, Tanaka et al. [70] developed a predictive tool based on timing measures like the timed-up-and-go test, single-leg stance, and walking speed, which provide only limited insights into movement dynamics relevant to LS. Das et al. [71] applied artificial neural networks and RF models to classify LS stages. However, their approach relies on the features extracted from functional tests like squats and single-leg stance, which are often challenging or even impractical for many older adults. Suyama et al. [72] used PCA for LS-related factor extraction. However, their classification was limited to broad categories like “somewhat LS” and “somewhat healthy”, resulting in ambiguous distinctions. Hashimoto et al. [73] employed KNN and Kinect-derived gait features. Their method relied on costly and less accessible equipment, and participants were classified only as LS or healthy, without distinguishing between LS stages. In contrast, our approach is based on a simple functional test using an accessible IMU, which can easily be integrated into everyday devices, making it a more practical and applicable solution for LS assessment.

Our approach is consistent with the methodologies used in similar studies on fall risk prediction and mobility assessment. Adeli et al. [61] used the ambient monitoring of gait to predict fall risk in individuals with dementia with the use of machine learning models to analyze longitudinal gait data. The study relied on sensor-based data collection and emphasized the importance of capturing biomechanical changes over time. Similarly, Agrawal et al. [74] leveraged wireless sensor insoles to measure real-time gait metrics, applying machine learning models for fall risk prediction. Both studies underscore the value of integrating wearable sensor technologies with advanced computational methods for detecting mobility impairments. In terms of methodology, these studies share key similarities with our approach, particularly in the use of sensor data and machine learning. In terms of methodology, these studies share key similarities with our approach, particularly in their reliance on sensor data and machine learning techniques. Adeli et al. [61] employed an MLP model to predict fall risk, leveraging its capacity to capture the nonlinear relationships within high-dimensional data. In contrast, Agrawal et al. [74] used traditional machine learning models, such as SVM, RF, and KNN, to classify fall risk levels based on real-time gait metrics collected from wireless insoles. However, we employed both traditional and deep learning models (MLP) to classify different LS stages. Like these studies, we focused on feature extraction from sensor data, including time-domain and frequency-domain features, to identify the movement patterns associated with mobility impairments. To address the class imbalance in their datasets, both studies employed data balancing techniques, including oversampling minority classes, akin to our use of the SMOTE method. This step ensures that machine learning models are not biased toward the majority class and enhances the ability to predict rare outcomes effectively. While some dimensionality reduction techniques have been explored in these studies, we employed both PCA and feature selection methods to optimize feature sets to improve model performance.

### 3.1. Dataset Handling

The experimental dataset consisted of acceleration and gyroscope data collected from 174 participants, including 125 older adults and 49 younger adults. The older participants were categorized into LS stages (LS-Stage 0, LS-Stage 1, LS-Stage 2) based on their corresponding GLFS-25 scores. Each participant performed the FTSTS test, generating kinematic data for each postural transition. Table 1 provides a detailed summary of the demographic distribution and group sizes. The recorded data from the FTSTS were collected using a single IMU and processed to segment SiSt, StSi, and SSS transitions. From these segments, 144 features were extracted across the time and frequency domains, including maximum, range, and RMS accelerations, jerk, and spectral edge frequencies in the ML, AP, SI, and resultant planes. The list of the extracted features alongside their descriptions can be found in the Supplementary Materials. The experimental setup involved splitting the dataset into training (80%) and testing (20%) sets. Several ML models were then trained using the training set, and their performance was compared and tested using the testing set.

### 3.2. Experiment Setting

This study included an MLP model, alongside six widely recognized traditional machine learning methods including K-nearest neighbors (KNN), support vector machine (SVM), decision trees (DTs), random forest (RF), gradient boosting classification (GB), and logistic regression (LR). The primary objective of this study was to categorize the different stages of LS based on the extracted time- and frequency-domain features from the three-axis accelerometer as well as to identify the optimal prediction model for the early detection of LS. To achieve this, we used the GridSearchCV technique from the sklearn library to determine the best hyperparameters for each machine learning method. The resulting optimal hyperparameters are presented in Table 3, which shows the optimum parameter configurations that contribute to enhanced model performance and accuracy. The careful selection of these features and hyperparameters ensures that the models are trained on the most discriminative aspects of the data, leading to better classification performance.

These hyperparameters were chosen based on a comprehensive grid search process, which systematically explored a range of values to identify the optimal configuration for each model. The grid search ensured that our models were fine-tuned to achieve the best possible performance on our dataset. For instance, in the RF model, the number of trees (n_estimators) and the depth of the trees (max_depth) are crucial for balancing bias and variance. Similarly, in the MLP model, the learning rate, batch size, and number of epochs are optimized to ensure efficient and effective learning. The L2_value parameter in MLP adds a penalty on the size of coefficients, helping to prevent overfitting, and the optimizer parameter specifies the optimization algorithm (e.g., Adam for adaptive moment estimation).

### 3.3. Addressing Class Imbalance

The original extracted feature set suffered from class imbalance, hindering the model training and resulting in suboptimal performance. To address this challenge, the SMOTE method was employed to balance the dataset and enhance model training, ensuring a balanced representation of all classes. Before augmentation, the dataset exhibited a glaring class imbalance, with the minority class (LS-Stage 1) comprising only 29 data points. This imbalance is a common issue in machine learning tasks and can significantly compromise the performance of predictive models, as they tend to favor the majority class due to its larger representation. However, the introduction of SMOTE brought about a remarkable transformation in the dataset. By generating synthetic instances for the minority class, SMOTE effectively increased its representation to 49 data points. This augmentation achieved two essential objectives. Firstly, it alleviated the class imbalance issue, ensuring that each LS stage had a more equitable presence in the dataset. Secondly, it increased the size of the dataset, which is particularly advantageous in terms of training and testing machine learning models.

By training various machine learning models on both the original and augmented datasets, we aimed to conduct a comprehensive performance analysis and assess how the balanced dataset impacted the model’s results. Table 4 presents a thorough comparison of the machine learning models’ performance on the original and augmented datasets. The results of the experimentation underscore the transformative impact of having a balanced dataset on the performance of different machine learning models when dealing with LS prediction. Initially, the models were trained on the imbalanced original dataset, leading to suboptimal outcomes across the board. For instance, the SVM model exhibited an accuracy of 0.26, a precision of 0.07, a recall of 0.26, and an F1 score of 0.11. These scores indicated a considerable struggle in distinguishing between the different LS stages.

However, the results changed considerably after using the augmented dataset. The SVM model, for instance, saw the highest surge in performance, with accuracy soaring to 0.67, precision reaching 0.67, recall attaining 0.67, and the F1 score leaping to 0.66. This transformation in performance was mirrored across multiple models. KNN, DTs, RF, GB, and MLP all exhibited notable improvements, emphasizing the substantial benefits of mitigating class imbalance through data augmentation techniques.

The results highlight the potential of both lightweight learning methods like RF, SVM, and KNN, as well as deep learning techniques such as MLP, for LS prediction. When provided with balanced training data, these models demonstrated substantial improvements in predictive accuracy, outperforming the other methods significantly.

**Figure 3:**
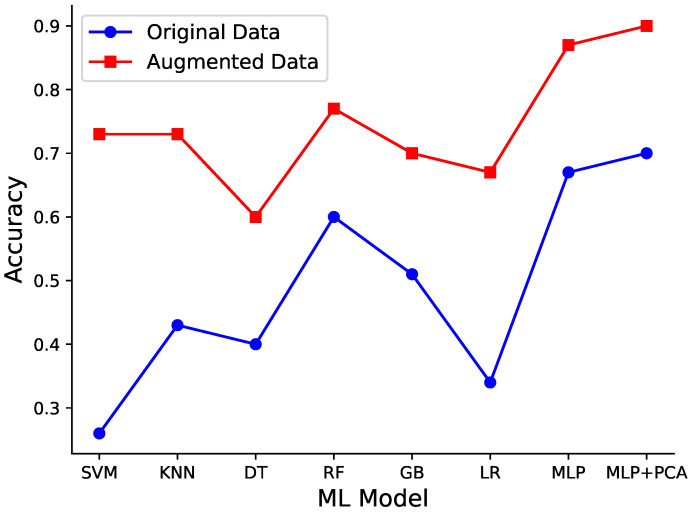
A line chart comparing the accuracy of eight different machine learning models on the original and augmented datasets. The augmentation significantly improves model performance, particularly for models like SVM, LR, and MLP.

The implications of this transformation are profound. As shown in Figure 3, the augmentation not only corrected the inherent bias favoring the majority class but also enhanced the model’s ability to classify various stages of LS. Additionally, the larger dataset generated by SMOTE introduced more diverse data points, thereby improving the models’ ability to generalize. This, in turn, contributes to the models’ overall performance and robustness when applied to real-world LS prediction scenarios.

### 3.4. PCA-Enhanced Model

The accuracy of the model using MLP, as reported in Table 4, when considering all the features as inputs to the model, was 0.91 and 0.87 on the training and test datasets, respectively. These results indicated the MLP’s competence in learning complex relationships within the feature set in making a more accurate prediction of LS compared to other machine learning models. However, a pivotal objective in model optimization is the reduction in complexity without compromising the performance of the prediction. Through dimensionality reduction, PCA condenses the feature space while preserving the most informative aspects of the features. In this context, incorporating PCA led to a significant improvement in the model’s accuracy. The MLP model, when trained on the PCA-transformed dataset, achieved an accuracy of 0.94 on the training data and 0.90 on the test data. The minimal differences between the training and test results suggested that overfitting was effectively mitigated through the implementation of L2 regularization and early stopping.

This improvement is multifaceted. It shows the model’s better ability to generalize from training to testing, reducing overfitting. Also, its improved accuracy indicates the model captures crucial features for LS prediction. By reducing the dimensionality of the input space, PCA can streamline the model’s focus on the most influential factors while discarding redundant information. Consequently, this refined feature set empowers the model to make more accurate predictions, optimizing its performance in the effective diagnosis and staging of locomotive syndrome. Although PCA helps in focusing on the most important features, contributes to the most variance in the data, and leads to a more robust and generalizable model by eliminating noise and irrelevant features, the transformed features (principal components) are linear combinations of the original feature set, which may not have a straightforward interpretation. This can make it challenging to understand the contribution of each original feature to the model’s predictions. To balance performance and interpretability, we selected a number of principal components that capture the majority of the variance while maintaining a manageable number of features. The trade-off between dimensionality reduction and interpretability is an important consideration, and future work may involve exploring methods to interpret the principal components in the context of the original features.

### 3.5. Streamlining Efficiency and Accuracy

In our experiments, we aimed to enhance efficiency and reduce computational complexity in LS prediction by integrating the SelectKBest feature selection method. This involved using mutual information as a criterion to identify the pivotal features within the dataset. The features were meticulously sorted based on their importance. The term ‘importance score’ refers to a measure quantifying the relevance of each feature in predicting LS. In this context, a higher importance score implies a greater contribution of the feature to the predictive accuracy of the model, while a lower score suggests a comparatively lesser impact. An iterative process was then initiated, gradually increasing the number of sorted features fed into the MLP model. This approach was designed to investigate the relationship between the number of features and the model’s performance. Features were systematically introduced in the order of their importance scores, allowing us to assess how the MLP model’s accuracy evolved with each feature’s inclusion.

**Figure 4:**
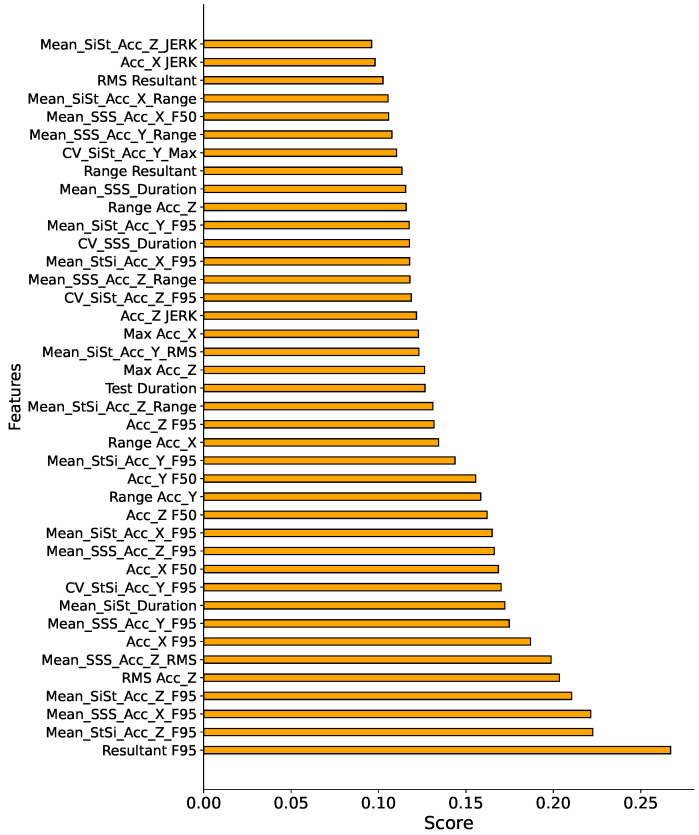
The top 40 most significant features ranked by their importance scores obtained through the SelectKBest feature selection method, reflecting the relevance of each feature to LS prediction.

The findings revealed that incorporating the top 40 features, as determined by their importance scores, resulted in an accuracy of 0.83. The top 40 features with the highest importance scores are shown in Figure 4. Despite the reduced feature set, the model managed to achieve a marginally lower level of accuracy compared to the more complex feature set, demonstrating the potential of this approach to streamline efficiency and reduce computational overhead without compromising prediction quality.

Furthermore, the experimental results demonstrated that the MLP’s performance reached a saturation point after the inclusion of 133 features, achieving an accuracy of 0.87. Beyond this point, the addition of more features did not yield any discernible improvement in accuracy. This observation suggests that the model effectively captured the relevant extracted features necessary for an accurate LS prediction within this subset of features.

### 3.6. Class Differentiation Analysis

**Figure 5:**
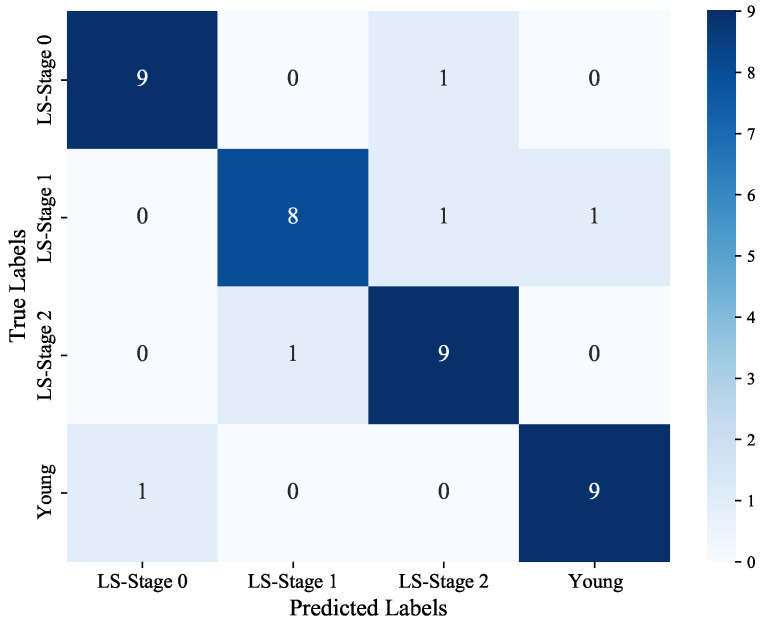
The confusion matrix showing the performance of the MLP+PCA model on the test dataset. The diagonal elements represent correct predictions, while off-diagonal elements indicate misclassifications of the model during the test phase.

The confusion matrix depicted in Figure 5 provides a comprehensive view of the model’s performance. This confusion matrix was created by enhancing the MLP architecture with PCA to improve its performance on the test set, achieving an accuracy of 0.90 on the augmented data. The diagonal elements of the matrix correspond to accurate predictions, displaying the number of instances correctly classified in each class. This indicates that the model effectively discerns instances within each class, particularly for LS-Stage 0, LS-Stage 1 and LS-Stage 2. Notably, the confusion between LS-Stage 1 and LS-Stage 2 is relatively prevalent, which may stem from the marginal differences between these classes that pose a challenge even for a sophisticated model. However, the model appears to exhibit some challenges in distinguishing between LS-Stage 0 and Young, as reflected in the off-diagonal elements of their respective rows. This in itself is not a concern. LS-Stage 0 symbolizes robust motor function. However, it is crucial for the model to effectively discern various LS stages in older adults, facilitating timely intervention and monitoring.

## 4. Conclusions and Future Work

In this study, we focused on using a single IMU with machine learning due to its compatibility with everyday devices like smartphones, enabling the convenient self-assessment of LS. We introduced an innovative approach by combining a machine learning model with an instrumented repeated STS test for LS classification. This work serves as the foundational step toward further research, paving the way for an LS classification model using everyday devices like smartphones. The integration of these machine learning models into a smartphone and a corresponding mobile application can establish a remote LS assessment and monitoring system, offering a promising avenue for practical implementation.

Classifying participants into different LS stages quantitatively could mark a breakthrough as it provides a structured, objective framework to track the progression of LS, allowing for targeted interventions at precise stages of decline. Unlike qualitative assessments, quantitative classification allows for finer distinctions, making it possible to detect even subtle changes in locomotive function. Unlike frailty and fall risk, which have been more widely studied, LS remains underexplored despite its specific importance in maintaining independence in older adults. Establishing a quantifiable method for assessing LS could thus help prioritize and tailor preventive care, delay the progression of musculoskeletal decline, and optimize resource allocation in geriatric health. By embedding LS classifications into electronic health records, healthcare providers can receive automated alerts when a patient’s LS score indicates a need for further assessment or intervention. This approach facilitates a more personalized care strategy, enabling clinicians to prioritize targeted interventions such as strength training and balance exercises based on specific LS stages. Additionally, quantifying LS can improve resource allocation by helping healthcare facilities anticipate care needs and optimize staffing. This quantitative approach offers a proactive means to manage the health of older adults, enhancing their quality of life and independence.

Despite the vast potential of machine learning and artificial intelligence, there have been limited explorations of their application in the diagnosis of LS. Recently, Takahashi et al. [69] looked into the feasibility of utilizing convolutional neural networks (CNNs) and foot pressure images for the detection of LS. While their findings yielded promising results, further investigation employing diverse machine learning techniques and different data modalities is warranted. In this study, seven distinct machine learning methods were explored, and their performances were compared. To address the challenge created by imbalanced data in different LS classes, an oversampling technique was employed. Among the models, the MLP model emerged as the most effective. To enhance efficiency, PCA was employed, resulting in the MLP+PCA model achieving remarkable accuracy (0.9), precision (0.92), recall (0.9), and F1 score (0.91), surpassing all other models. These findings highlight the significant potential of the MLP+PCA model for accurate LS classification.

The findings of this study should be considered in light of its limitations. First, the classification of LS stages did not account for demographic information, such as sex and age, which may or may not influence LS and its progression. While we are not certain of the impact of these demographic factors, further investigation would be valuable in clarifying any potential associations. Additionally, the small sample size of the participants limits the generalizability of our findings. Larger cohorts could capture a wider range of clinical presentations, increasing the robustness and relevance of the classification model across diverse demographics. The GLFS-25 questionnaire, developed in Japan, includes some items that may not be universally applicable to Australian participants. For instance, questions about public transport use may not fully resonate with those in Australia’s rural or regional areas, where public transport is less common. This cultural difference may impact the sensitivity and specificity of the LS classification, as certain aspects of daily life in Australia are not reflected in the questionnaire. In this study, SMOTE was employed to address the data imbalance, but it does have limitations. Oversampling can sometimes result in overfitting, as the synthetic samples may not perfectly capture real-world clinical complexities. SMOTE also does not account for the correlations between features, potentially distorting the dataset by introducing patterns that do not fully reflect clinical cases. Expanding the cohort size in future studies will help to address the issue of imbalanced classes and enhance the generalizability of our results [75].

In future studies, we plan to adapt the GLFS-25 questionnaire to fit Australia’s cultural context. This will involve reviewing existing items to ensure they are relevant for the Australian population. We may also seek feedback from local healthcare professionals and geriatric care experts. For example, one question in the GLFS-25 asks if participants often use public transport. However, public transport is less common in rural and regional Australia compared to Japan. By making these adaptations, we aim to improve the questionnaire’s acceptance and effectiveness among Australian clinicians and patients. To improve the generalizability of our findings, we will expand our participant cohort by recruiting a more diverse range of individuals across various age groups, socioeconomic backgrounds, and geographical locations. This will help us address data imbalance issues at each locomotive syndrome stage and ensure that our results are reflective of the broader population. Moreover, we will compare our results with other established physical performance tests commonly used in geriatric assessments. This comparative analysis will provide a more comprehensive understanding of locomotive function and enhance the validity of our findings. We also plan to correlate the features from the FTSTS test with additional physical assessments that directly measure leg strength and balance control, such as muscle strength and postural balance assessment tests. This will allow us to better understand how variations in the FTSTS test features relate to these specific physical abilities that are affected by LS. Finally, we will evaluate the accuracy and relevance of our machine learning models by comparing them with clinician-scored LS assessments. This will help with validating the technology-driven approach and highlighting any discrepancies that may warrant further investigation. Through these efforts, we aim to enhance the validity and applicability of our approach for LS diagnosis and management in everyday clinical practice, ultimately improving the quality of care for older adults in Australia.

## Figures and Tables

**Table 1:** A summary of participants’ demographic characteristics.

	Participants (#)	Sex (M/F)	Age (Years)
Non-LS	47	21/26	71.4±6.1
LS-Stage 1	29	8/21	72.3±8.6
LS-Stage 2	49	19/30	76.1±11.3
Young	49	30/19	27.04±7.1

**Table 2:** The parameter ranges explored during the grid search for each machine learning model.

Classifier	Parameter	Values
SVM-linear	C	[0.1, 1, 10, 100, 1000, 10000]
SVM-rbf	C	[0.1, 1, 10, 100, 1000, 10000]
	gamma	[0.001, 0.01, 0.1, 1, 10]
KNN	n_neighbors	[1, 2, ., 50]
	weights	[‘uniform’, ‘distance’]
DT	max_depth	[1, 2, ., 50]
	criterion	[‘gini’, ‘entropy’]
RF	n_estimators	[50, 100, 200, 400, 600, 800, 1000, 1200]
	max_depth	[None, 5, 10, 20, 30, 50]
	min_samples_split	[2, 5, 10]
	min_samples_leaf	[1, 2, 4]
	bootstrap	[True, False]
GB	n_estimators	[50, 100, 200, 400, 600, 800, 1000, 1200]
	learning_rate	[0.1, 0.01, 0.001]
	max_depth	[None, 5, 10, 20, 30, 50]
	min_samples_split	[2, 5, 10]
	min_samples_leaf	[1, 2, 4]
LR	C	[0.1, 1, 10]
	penalty	[‘L1’, ‘L2’]

**Table 3:** The optimal hyperparameters found for each machine learning model using GridsearchCV. These hyperparameters were selected based on their performance during validation in order to enhance the model’s accuracy.

Classification Model	Parameters
SVM	{‘C’: 10, ‘kernel’: ‘rbf’, ‘gamma’: 0.001}
KNN	{‘n_neighbors’: 5, ‘weights’: ‘distance’}
DT	{‘criterion’: ‘entropy’, ‘max_depth’: 12}
RF	{‘bootstrap’: False, ‘max_depth’: None, ‘min_samples_leaf’: 2,
	‘min_samples_split’: 2, ‘n_estimators’: 50}
GB	{‘learning_rate’: 0.01, ‘max_depth’: 5, ‘min_samples_leaf’: 1,
	‘min_samples_split’: 10, ‘n_estimators’: 600}
LR	{‘C’: 0.1, ‘penalty’: ‘L2’}
MLP	{‘learning_rate’: 0.001, ‘epochs’: 200, ‘batch_size’: 64,
	‘L2_value’: 0.001, ‘optimizer’: Adam}

**Table 4:** A comparison of various machine learning models’ performance on the original dataset versus the augmented dataset created using SMOTE.

Evaluation Metric	Original Data								Augmented Data							
	**SVM**	**KNN**	**DT**	**RF**	**GB**	**LR**	**MLP**	**MLP+PCA**	**SVM**	**KNN**	**DT**	**RF**	**GB**	**LR**	**MLP**	**MLP+PCA**
Accuracy	0.26	0.43	0.40	0.60	0.51	0.34	0.67	0.70	0.73	0.73	0.60	0.77	0.70	0.67	0.87	0.90
Precision	0.07	0.31	0.40	0.54	0.50	0.24	0.67	0.73	0.80	0.80	0.67	0.74	0.73	0.70	0.84	0.92
Recall	0.26	0.43	0.40	0.60	0.51	0.34	0.67	0.70	0.73	0.73	0.60	0.76	0.70	0.67	0.88	0.90
F1 score	0.11	0.36	0.39	0.56	0.50	0.28	0.66	0.71	0.74	0.74	0.62	0.74	0.70	0.67	0.86	0.91

## Data Availability

All data supporting this study are available upon request.

## Supplementary Materials

The following supporting information can be downloaded at: https://www.mdpi.com/article/10.3390/s24237727/s1, Table S1: List of 144 extracted features from the 3-axis accelerometer and their corresponding descriptions.

## Abbreviations

The following abbreviations are used in this manuscript:

Acc	Acceleration
ADL	Activities of Daily Living
AP	Anterior–Posterior
CV	Coefficient of Variance
CNN	Convolutional Neural Network
DT	Decision Tree
FTSTS	Five-Time Sit to Stand
GB	Gradient Boosting
GLFS-25	Geriatric Locomotive Function Scale 25
IMU	Inertial Measurement Unit
JOA	Japanese Orthopaedic Association
KNN	K-Nearest Neighbors
LR	Logistic Regression
LS	Locomotive Syndrome
Max	Maximum
Min	Minimum
MLP	Multilayer Perceptron
ML	Mediolateral
PCA	Principal Component Analysis
QOL	Quality of Life
ReLU	Rectified Linear Unit
RF	Random Forest
RMS	Root Mean Square
SEF50	Spectral Edge Frequency 50
SEF95	Spectral Edge Frequency 95
SI	Superior–Inferior
SiSt	Sit–Stand
SSS	Sit–Stand–Sit
SMOTE	Synthetic Minority Oversampling Technique
StSi	Stand–Sit
SVM	Support Vector Machine
TUG	Timed Up and Go
